# Fear of Treatments Surpasses Demographic and Socioeconomic Factors in Affecting Patients With Breast Cancer in Urban South Africa

**DOI:** 10.1200/JGO.2015.002691

**Published:** 2016-06-22

**Authors:** Sarah Rayne, Kathryn Schnippel, Cynthia Firnhaber, Kathryne Wright, Deirdre Kruger, Carol-Ann Benn

**Affiliations:** All authors, University of the Witwatersrand; and Kathryn Schnippel and Cynthia Firnhaber, Right to Care, Johannesburg, South Africa.

## Abstract

**Purpose:**

Breast cancer is the most common cause of cancer in women in South Africa, and often patients present late. There is little understanding of the psychosocial stresses affecting women with breast cancer in Africa.

**Methods:**

A questionnaire was distributed to 263 patients with breast cancer at two sites (one government and one private facility) in Johannesburg. Self-reported levels of fear were recorded on summative scales and their relationship to demographic variables assessed through univariable and multivariable modified Poisson regression.

**Results:**

Fears related to treatments and prognosis, particularly radiation, loss of hair, and loss of breast, were far stronger than those related to socioeconomic barriers. Relative risk (RR) of most fears was higher in women younger than age 40 years, including treatment affordability (RR, 1.80; 95% CI, 1.26 to 2.56), hair loss (RR, 1.48; 95% CI, 1.12 to 2.95), and surgery (RR, 1.31; 95% CI, 1.02 to 1.68). Difficulty taking time off work predicted fear of job loss (RR, 2.59; 95% CI, 1.59 to 4.21) and missing appointments because of transport (RR, 2.46; 95% CI, 1.52 to 3.96) or family commitments (RR, 2.46; 95% CI, 1.52 to 3.96). Women with dependents and black women were more afraid of dying (RR, 1.73; 95% CI, 1.03 to 2.90; and RR, 1.79; 95% CI, 1.33 to 2.24, respectively); however, socioeconomic status in this sample was a strong confounder of race and explained most of the racial differences in levels of fear.

**Conclusion:**

The most significant fears around breast cancer were related to treatment modalities and adverse effects rather than transport, financial, or work concerns. Young age and job insecurity were predictive of increased fears. Education about treatments has a key role to play in improving access to breast cancer care in South Africa.

## INTRODUCTION

Breast cancer is the most common form of cancer to affect women in South Africa and in 2013 was responsible for 20.8% of female cancers and more than 10% of the entire cancer burden.^1^ The National Cancer Registry (most recent report, 2009) reports an age-adjusted incidence of 27.6 per 100,000, equating to a lifetime risk of 1 in 33 women.^1^

In developed countries, advances in the management of breast cancer over the last 50 years, including screening and early detection programs, have led to an increase in survival rates. Early-stage breast cancer has a survival of > 95%;^2^ however, late-stage disease continues to be responsible for most cancer deaths, through metastatic progression.

Optimal treatment of breast cancer and increased survival rely not just on early detection but on early and continued presentation at a center capable of treating the disease. Thus, health care disparities will affect a patient’s outcome.^3^ In trying to understand these disparities and their effect on outcome, studies often look to socioeconomic factors, including education, health insurance status, work, and transportation.^4,5^ These factors may provide physical barriers in accessing adequate health care. Physical barriers can also become a source of psychosocial stress for the patient, causing fears that can also affect timely presentation, treatment, and patient choices about their breast cancer care.^6^ In addition to physical barriers to access of care and fears related to sociodemographic factors, further stress can be associated with knowledge (or lack of knowledge) of treatment modalities and their associations and adverse effects.

Understanding the fears associated with breast cancer in South Africa is important in improving care and enabling and encouraging all women to access treatment earlier. By interviewing women accessing services in both government and private facilities in Johannesburg, we sought to determine the fears experienced by all women with a diagnosis of breast cancer, allowing identification of important universal areas for education and improved care provision.

## METHODS

This dual-center study included patients with and without medical aid in Johannesburg, South Africa. Medical aid is the system of health insurance in South Africa bought by the end-user that allows access to private facilities. Patients without medical aid or with poor coverage are seen in government-funded facilities at low or no cost. The government clinic is based within a provincial government tertiary hospital and manages approximately 350 new breast cancer diagnoses each year. Only 6% of these patients have medical insurance (unpublished local data). The private facility sees private- or medical insurance–funded patients and manages approximately 400 new breast cancer cases per year.

From November 2011 to May 2013, patients undergoing treatment of breast cancer in each center were recruited to complete a questionnaire. The questionnaire was distributed to consenting patients attending for diagnosis, operation, or follow-up over a 21-month period. Each patient was offered the choice to complete the questionnaire alone or with the aid of an assistant, who could translate into the patient’s preferred language. The study received ethical approval from the Human Research Ethics Committee of the University of the Witwatersrand (M111121).

The first part of the questionnaire included sections on demographics, socioeconomic status, educational background, work, transport, and religious affiliations. The second part of the questionnaire used a summative scale of 0 (no fear) through 5 (fear under control) to 10 (very fearful) to determine the patients’ feelings of fear and concern at diagnosis about issues related to their care, social circumstances, or prognosis.

Patient characteristics were described using frequencies and proportions. Age was categorized as age 40 years and younger or age 41 years and older. Having dependents was compared with not having dependents. Social status was categorized as being married or unmarried, the latter of which included women who were single, divorced, or widowed. Women who were employed by others or considered themselves self-employed were compared with women who were unemployed or retired. Education was grouped according to highest level of formal schooling completed: primary, secondary, or tertiary. Reported race was collapsed into black and nonblack categories, including women who indicated white, Indian (Asian), colored, and mixed races. Women who indicated that they sometimes or always had difficulty with transport or taking time off from work were compared with women who indicated they seldom or never had those difficulties. Two-sided differences of proportions were compared using Pearson’s χ^2^ test. A *P* value < .05 was considered statistically significant.

Patients who did not have a confirmed diagnosis of cancer were excluded from the analysis of fears (n = 4). Responses were categorized as fearful (score > 5) or not fearful. Modified Poisson regression with robust error estimation^7^ was applied to the data to estimate the relative risk (RR) of being fearful according to patient characteristics; RRs and a 95% CIs are presented. Univariable and multivariable results adjusted for the patient characteristics described above are presented. All statistical analyses were done in Stata v13.1 (College Station, TX).

## RESULTS

A convenience sample of 263 patients was included. Patient age ranged from 18 to 86 years, with a median of 52 years (interquartile range, 44 to 62), with 41 patients who were age 40 years or younger at diagnosis (16%). Table 1 lists patient characteristics grouped as either private hospital or government hospital and shows that government patients were more likely to be black (*P* < .001), single, and living with others. They were less likely to be employed when compared with private facility patients (52% *v* 73%; *P* = .001). Significantly more private facility patients had a tertiary education (53% *v* 13%, *P* < .001), and 36% of government patients attained primary education only compared with 6% from the private facility. Access to cell phones was extremely high in both groups (97% private facility, 95% government), but computer and internet usage was significantly higher in private patients (87% *v* 39%, *P* < .001).

**Table 1:**
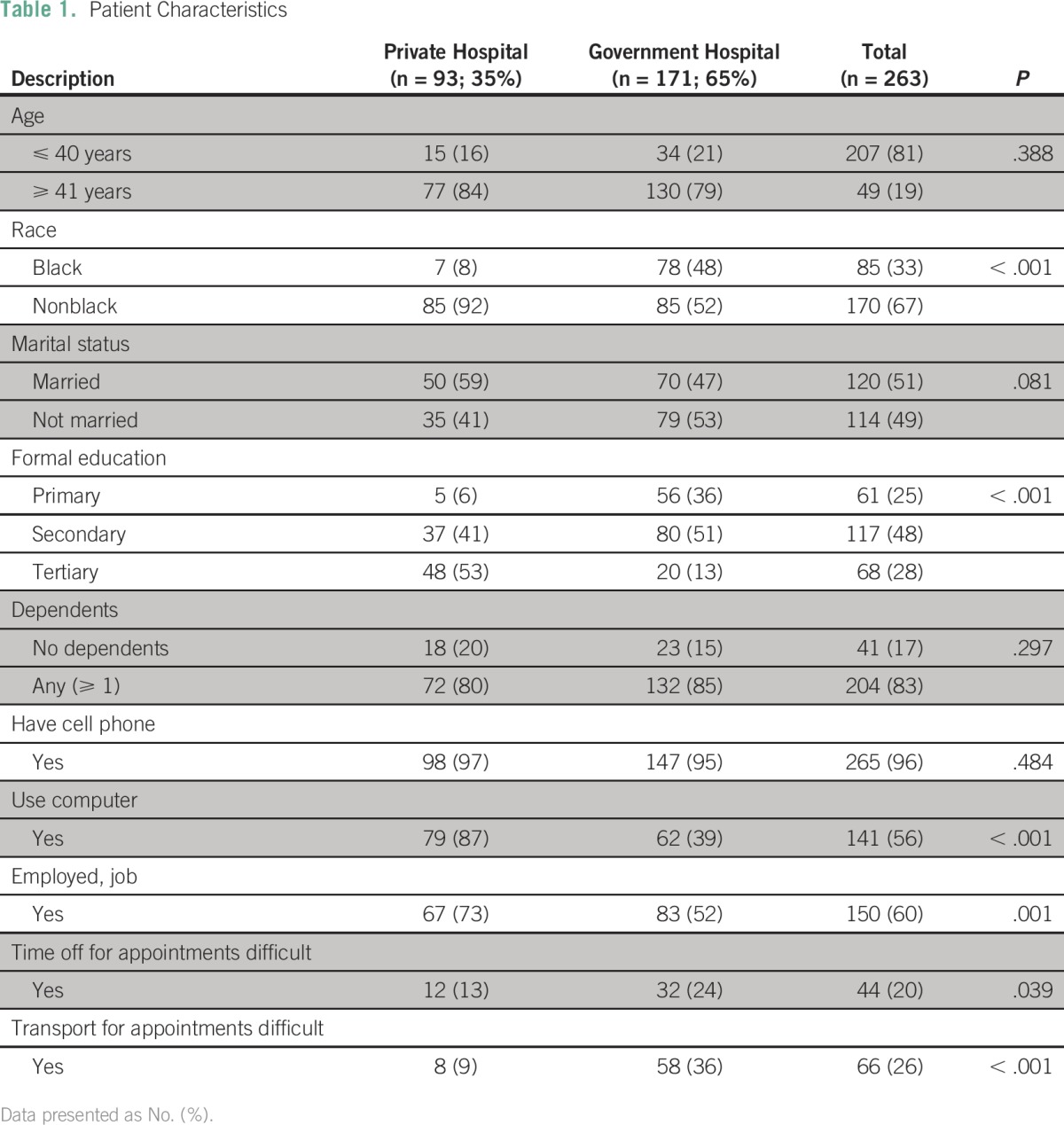
Patient Characteristics

### All Fears

**Fig 1:**
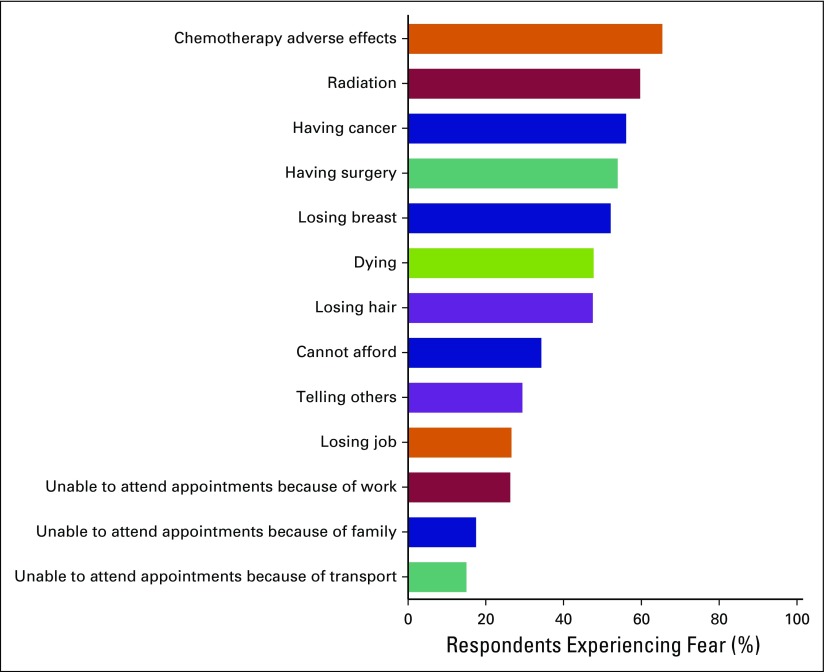
The percentage of women categorized as fearful (fear score > 5) in each described fear.

Figure 1 illustrates how many patients with a confirmed cancer diagnosis (n = 259) were fearful (score > 5) for each domain included in the questionnaire. The overall pattern demonstrates that fears related to treatments and prognosis were far stronger than those related to socioeconomic barriers. The most common treatment-related fears were related to chemotherapy adverse effects (65.4%), followed by radiation and surgery (59.7% and 53.9%, respectively). Although some patients feared both surgery and breast loss, the differences between the women who feared surgery and those who feared breast loss was statistically significant (*P* < .001), as were women who feared surgery and women who feared radiation (*P* < .001), using the Pearson’s χ^2^ test. Concerns over ability to attend appointments for reasons including transport and work were the lowest.

### Relative Risks of Different Fears

**Fig 2:**
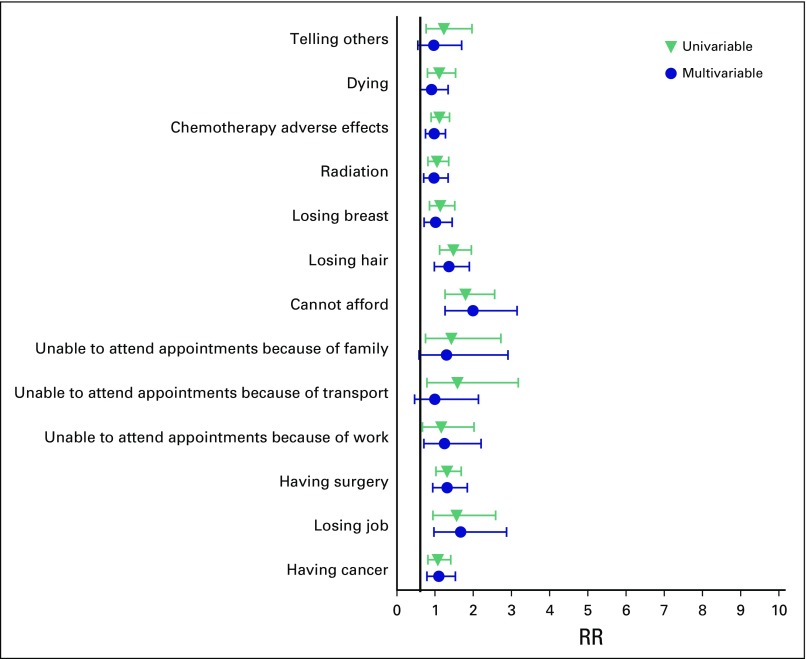
The relative risk (RR) of fears in women age 40 years or younger shown relative to those older than age 40 years.

The RRs of fearfulness for women younger than age 40 years were uniformly higher than for older women (Fig 2). This was most pronounced in their worry over affording all appointments and treatments (RR, 1.80; 95% CI, 1.26 to 2.56). Young women also had an increased likelihood of treatment-related fears, significantly in fear of hair loss (RR, 1.48; 95% CI, 1.12 to 2.95) and fear of surgery (RR, 1.31; 95% CI, 1.02 to 1.68).

In women of all ages, difficulty with taking time off work (a relative measure of job insecurity) had increased likelihood of fear about job loss (RR, 2.59; 95% CI, 1.59 to 4.21), and women were more likely to fear missing appointments either because of transport (RR, 2.46; 95% CI, 1.52 to 3.96) or because of family commitments (RR, 2.46; 95% CI, 1.52 to 3.96). They also feared the adverse effects of chemotherapy more (RR, 1.30; 95% CI, 1.07 to 1.58). A tertiary education was protective against fear of job loss (RR, 0.46; 95% CI, 0.24 to 0.92) and not attending appointments because of transport and family (RR, 0.20; 95% CI, 0.07 to 0.58; and RR, 0.33; 95% CI, 0.14 to 0.75, respectively).

**Fig 3:**
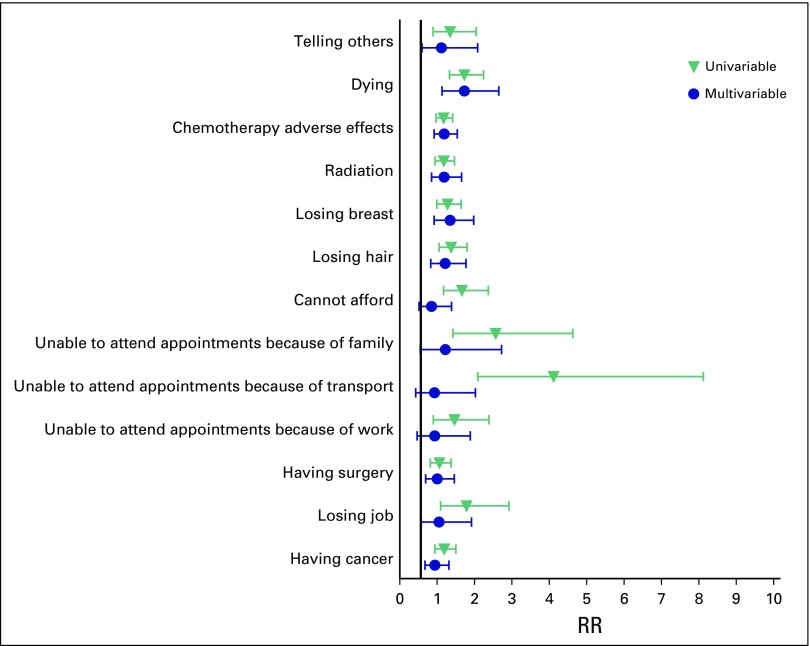
The relative risk (RR) of fears in women of black race shown relative to all other women in the study.

**Fig 4:**
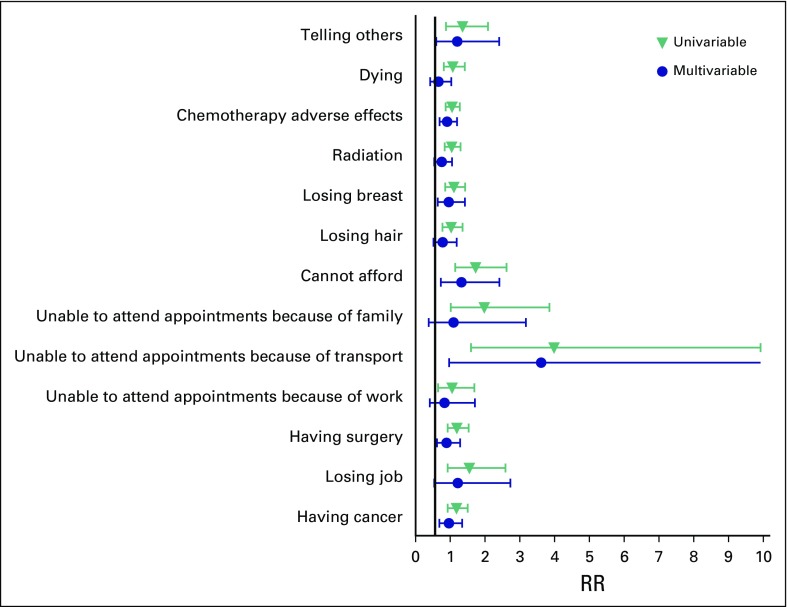
The relative risk (RR) of fears in women at public sector hospital shown relative to women at private sector hospital.

The RR of fear of dying had a median of 5 and the widest spread of any of the fears, with an interquartile range of 1 to 9; 47.7% of participants indicated they were fearful of dying (a score > 5). Women with dependents were 1.73 times more likely to be afraid of dying (95% CI, 1.03 to 2.90), as were black women (RR, 1.79; 95% CI, 1.33 to 2.24). Figure 3 shows that black women experience much more fear toward the physical barriers to care: they were much more afraid of missing appointments because of transport problems (RR, 4.11; 95% CI, 1.42 to 8.12) or because of family commitments (RR, 2.56; 95% CI, 1.42 to 4.62) and worried they could not afford to get cancer (RR, 1.66; 95% CI, 1.17 to 2.37). These results are similar to those experienced by women in the public hospital (Fig 4).

**Fig 5:**
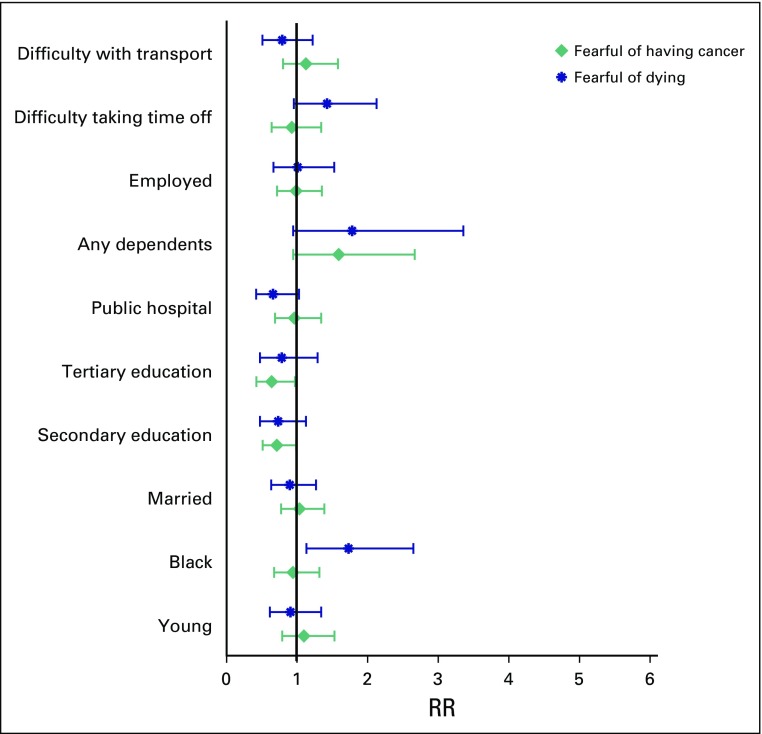
Multivariable analysis of influences on the fears of affordability of appointments and treatment and dying. RR, relative risk.

In adjusted regression, where patient characteristics were held constant (Fig 5), black race remained a significant predictor of fear of dying (adjusted RR, 1.73; 95% CI, 1.13 to 2.64). Socioeconomic characteristics such as employment, use of the government hospital, and computer use were not statistically significant predictors of fear of dying. Although being young, black, in a government hospital, or with transport difficulties increased the fear of affordability of treatments, none remained significant in the multivariable adjusted model.

## DISCUSSION

In women with a diagnosis of breast cancer, this study found that breast cancer treatment and its adverse effects, including the universal fears of cancer and dying, were associated with increased fear at diagnosis, but socioeconomic factors, including transport, work, and financial issues, were not. Affordability of cancer treatment was the only socioeconomic factor that was associated with an increased level of concern.

Studies have found that fear can be important in determining delay to treatment.^8-11^ How patients are treated also seems to influence if they continue to attend for care, whether by their physicians^12,13^ or because of adverse effects.^10,14,15^ However, the relative contribution of these factors to fear associated with a diagnosis has not been determined. The new findings from this study suggest that physical obstacles may represent less of a barrier to care than psychological fears associated with treatments in our urban South African setting.

Within this sample, different populations emerged, each with unique combinations of concerns. Like many other studies,^16,17^ we found a significant relationship between younger age and higher cosmetic fears around hair loss, although not loss of the breast. This lack of fear around mastectomy may reflect the options of reconstruction and breast-conserving surgery. Younger women also face increased fears over affordability of treatment, having surgery, and job insecurities. These differences reflect the pressures these women face in maintaining income after a diagnosis. The loss of hair could also be concerning in this light, as it would publicize cancer therapy to employers and colleagues. Studies that have looked at the psychological needs of young women have found that younger patients viewed themselves as different from older patients, with problematic work and relationship issues, fertility decisions, and poor support when transitioning into survivorship.^18,19^ Our findings are consistent with this and highlight the distinctive support needs of this young group.

In addition, this study highlighted other groups with less psychological resilience around breast cancer treatments, including those with job insecurities (increased fear of job loss) and those with dependents (fear of dying), whose additional supportive needs may be overlooked. The near-universal fear of cancer, with all its negative connotations, was present in increasing measure in those with dependents. In an economic center such as Johannesburg, members of the working population are often held responsible for household dependents in addition to supporting an extended family and homesteads in rural areas. It is possible that the demands of close caregiving to family, the economic demands of dependents near and far, and the unexpected nature of cancer can add significant psychological stress to such patients.

The highest-scoring fear was in relation to possible radiation treatment. This mirrors other studies that show women have a nonspecific fear of radiation^5,20^ and possibly fear its relationship to causing cancer. These findings highlight the important role of education of the presymptomatic woman (essentially every woman) in how breast cancer is not just diagnosed but also treated. Education has been shown to reduce fears and improve attendance in diverse global populations,^21-23^ including recent successes of HIV/AIDS community education in sub-Saharan Africa, where radio and television soap operas have been used to highlight diseases such as HIV/AIDS with measurable effect.^24^ The contribution of recent high-profile breast cancer survivors in South Africa can also be used as an opportunity to introduce public discussion of treatment options and successes in breast cancer care.^25^

The current study indicated an extremely high ownership of mobile phones in the population studied (89%). This, coupled with the increasing use of internet through smartphone connectivity, allows eHealth (using communication technology for health) and mHealth (using mobile communication technology to deliver health care) strategies to be used for education. Already, young patients in South Africa access health information for themselves and family through smartphone internet access,^26^ and this has been shown to modify health behavior, if not knowledge, in a South African patient population.^27^ These forms of education can complement more traditional mass-education initiatives, which have been used in breast cancer awareness in low-income, low-awareness areas of the world^28,29^ and in South Africa, with layering of messages of education through multimedia.^24^ This new finding that breast cancer treatments are a source of fear and potential barrier to care means that introducing management options and expectations into the dialogue of breast cancer awareness and screening should be a practical application after this study for cancer care in South Africa.

The fear of dying in black women was moderated by attendance at a government hospital. This may be a reflection of good social support, closer ties to community, and in particular the presence of survivor-navigators in the government hospital, funded by a nongovernmental organization. Other studies have also found that having counsellors or navigators present reassures patients and helps them negotiate the complex medical system, and they are then likely to receive recommended standard treatment.^30^ In addition, navigators are able to identify and resolve intrapersonal (defined as beliefs, knowledge, attitudes, and socioeconomic) barriers that affect patients.^31^ Securing funding to maintain and increase the number of available navigators is a challenging undertaking in our resource-limited environment; however, the potential future outcome of improved patient care highlights the necessity.

Previous studies have shown strong relationships between disparities of access and response to breast cancer care for different races internationally and in South Africa.^14,32^ Studies from Africa or of African migrants often rely on using cultural beliefs and fears to explain these barriers to care,^4-6,8,11,32,33^ whereas in this study we found that although there were differences between races in their levels of fear, these were related to inherent socioeconomic disparities rather than cultural responses. Black women were more likely to fear hair or breast loss and more likely to experience concerns over socioeconomic obstacles. However, because of the legacy of previous economic/political policies in the country, there remains a significant association between race and socioeconomic status. These socioeconomic factors were strong confounders of race in this study and, on multivariable analysis, most of the race-fear relationships weakened, demonstrating few inherent racial differences but inherent strong educational and economic drivers of fear. These data provide preliminary support for questioning the existence of culturally bound reasons for delay and failure in breast cancer treatment and point to more holistic concerns shared by women of all races in all countries.

In all countries, but particularly in post-apartheid South Africa, relying on factors such as race to predict fears, attitudes, and access is problematic. These data confirm the questioning of the influence race alone has on fears around breast cancer and receptiveness to treatment and, in fact, whether universal concerns over welfare and fear around treatment and death transcend race or culture. Health surveys among other seemingly homogeneous groups show great diversity of language, education, diet, and health behaviors,^34^ and therefore assuming help-seeking behavior or barriers will be standardized in a racial group should be treated with caution.

For practical reasons, an opt-in invitation method was used in this study, and no information is available for those who declined to complete the questionnaire. Although translation was available, there may be a selection bias toward English-speaking patients of all races, and this may confound some of the variables, particularly around education and employment. Therefore, the extent to which this can be generalized to all patients with breast cancer is unknown. An additional consideration and scope for further work is that the study was purely quantitative in its approach. Examination of the full scope of fears and attitudes of patients through interviews would increase the range of our understanding of the patient’s experience.

The study was cross-sectional in nature, and although women were asked to recall the fear experienced at diagnosis, in recollection some of these fears may have been moderated or increased for women who had already started treatment. In addition, a further limitation as with many studies of breast cancer in sub-Saharan Africa is that this sample is taken from patients attending an urban specialist center. Although they would have vital and valid responses for this study, particularly regarding access and barriers to care, it is impossible to target patients with breast cancer who fail or refuse to present to medical services at any point in their care. However, many of the patients in this study would be presenting in the later stages of the disease for final palliative care of fungating wounds or terminal disease symptoms. More studies are required to relate medical details, including staging at presentation, to psychosocial and socioeconomic characteristics.

In conclusion, studies on barriers to cancer care in underserved populations have often focused on the physical barriers that prevent patients from accessing care. Our results show that women are far less fearful of how they will negotiate life during treatment (including work, finances, and family commitments) than they are of the planned treatments. Young patients are particularly vulnerable to increased fear when approaching treatment of breast cancer, and this group should be targeted with specific support. For all women, the public message of breast awareness should include education about breast cancer treatment and emphasize the importance of treatment for survival.
